# Oncologic Outcomes of Radical Prostatectomy and High-Dose Intensity-Modulated Radiotherapy with Androgen-Deprivation Therapy for Relatively Young Patients with Unfavorable Intermediate-Risk Prostate Adenocarcinoma

**DOI:** 10.3390/cancers13071517

**Published:** 2021-03-25

**Authors:** Szu-Yuan Wu, Shyh-Chyi Chang, Chang-I Chen, Chung-Chien Huang

**Affiliations:** 1 Department of Food Nutrition and Health Biotechnology, College of Medical and Health Science, Asia University, Taichung 413, Taiwan; szuyuan@tmu.edu.tw; 2 Big Data Center, Lo-Hsu Medical Foundation, Lotung Poh-Ai Hospital, Yilan 256, Taiwan; 3 Division of Radiation Oncology, Lo-Hsu Medical Foundation, Lotung Poh-Ai Hospital, Yilan 256, Taiwan; 4 Department of Healthcare Administration, College of Medical and Health Science, Asia University, Taichung 413, Taiwan; 5 Cancer Center, Lo-Hsu Medical Foundation, Lotung Poh-Ai Hospital, Yilan 256, Taiwan; 6 Graduate Institute of Business Administration, Fu Jen Catholic University, Taipei 242062, Taiwan; 7 Centers for Regional Anesthesia and Pain Medicine, Taipei Municipal Wan Fang Hospital, Taipei Medical University, Taipei 110, Taiwan; 8 Department of Urology, Lo-Hsu Medical Foundation, Lotung Poh-Ai Hospital, Yilan 256, Taiwan; mork2747@gmail.com; 9 Faculty of Medicine, National Yang-Ming University School of Medicine, Taipei 11221, Taiwan; 10 International Ph.D. Program in Biotech and Healthcare Management, Taipei Medical University, Taipei 110, Taiwan; dcchen@tmu.edu.tw; 11 Department of Health Care Administration, College of Management, Taipei Medical University, Taipei 110, Taiwan

**Keywords:** radical prostatectomy, intensity-modulated radiotherapy, unfavorable intermediate risk, survival, prostate cancer

## Abstract

**Simple Summary:**

Scarce reports have evaluated oncologic outcomes in relatively young men with unfavorable intermediate-risk prostate cancer (UIR-PC) receiving radical prostatectomy (RP) or high-dose intensity-modulated radiotherapy (IMRT). After a literature review, we present the leading and largest head-to-head propensity score-matched study to examine all-cause death, biochemical failure (BF), locoregional recurrence (LRR), and distant metastasis (DM) in relatively young men with UIR-PC undergoing RP or high-dose IMRT. After adjustment for confounders, RP was found to be superior to high-dose IMRT in terms of the patients’ overall survival, BF, LRR, and DM.

**Abstract:**

Purpose: To estimate the oncologic outcomes of radical prostatectomy (RP) and high-dose intensity-modulated radiotherapy (IMRT) with short-term androgen-deprivation therapy (ADT) in relatively young men with unfavorable intermediate-risk prostate cancer, as defined by the National Comprehensive Cancer Network (NCCN-UIR-PC). Patients and Methods: We enrolled relatively young men (≤65 years) from the Taiwan Cancer Registry who had been diagnosed as having NCCN-UIR-PC and who had received RP or high-dose IMRT (at least ≥72 Gy) with short-term ADT (4–6 months). After propensity score matching of the confounders, Cox proportional regression was used to model the time from the index date (i.e., date of diagnosis) to all-cause death, biochemical failure (BF), locoregional recurrence (LRR), and distant metastasis (DM). Results: The corresponding adjusted hazard ratios (95% confidence intervals) of the risk of all-cause death, BF, LRR, and DM were 2.76 (1.36–5.60, *p* = 0.0050), 2.74 (1.72–4.84, *p* < 0.0001), 1.28 (1.09–1.90, *p* = 0.0324), and 2.11 (1.40–4.88, *p* = 0.0052), respectively. Conclusions: RP is superior to high-dose IMRT with short-term ADT in terms of oncologic outcomes for relatively young men with UIR-PC.

## 1. Introduction

An estimated 1,300,000 new cases of prostate cancer (PC) are reported annually worldwide, making it the second most common cancer diagnosis in men [1]. The Surveillance, Epidemiology, and End Results program indicated that between 2010 and 2015, the incidence of low-risk PC decreased while that of intermediate-risk, high-risk, and very high–risk PC increased in relatively young men in the United States [2]. Thus, the proportion of men with very high–risk, high-risk, and intermediate-risk PC is more than that with low-risk PC (approximately 10%) [1,2,3]. Findings in the United States are similar to those based on the Taiwan Cancer Registry database (TCRD) [3]. PC is the 6th leading cause of cancer-related death and the 5th most frequently diagnosed cancer among men in Taiwan [3]. Based on the National Comprehensive Cancer Network (NCCN) risk classification, intermediate-risk PC is the most common PC diagnosis worldwide as well as in Taiwan [1,2,3], and thus, the oncologic outcomes of different treatments for men with intermediate-risk PC, especially those with a longer life expectancy, are valuable for decision-making by clinicians and patients.

The initial management of PC in patients who are newly diagnosed must consider the risk of progression to a metastasis, potentially lethal disease and the prolonged natural history of the disease [4]. The initial evaluation should include clinical staging according to a digital rectal examination by an well-trained clinician to assess the extent of PC, Gleason score or grade group based on the initial biopsy, pretreatment serum prostate-specific antigen (PSA) level, and number of biopsy cores and extent of cancer involvement [4]. This allows men with PC to be stratified into NCCN risk classification according to the PC characteristics [4,5,6,7]. Consensus is lacking regarding the optimal risk stratification system for treatment selection for PC [8]. However, we used the risk categories defined by the NCCN in Taiwan, which have been used in the guidelines of the American Society for Radiation Oncology (ASTRO), Society of Urologic Oncology and American Urological Association (AUA) and have been largely endorsed by the NCCN, the American Society of Clinical Oncology, and all physicians in Taiwan [4,5,6,7].

Moreover, there is a lack of consensus regarding the optimal treatment choice for unfavorable intermediate-risk (UIR) PC. The treatment of choice for UIR-PC defined according to NCCN guidelines (NCCN-UIR-PC) is either radical prostatectomy (RP) or radiotherapy (RT) with short-term androgen-deprivation therapy (ADT) of 4–6 months, according to the NCCN guidelines for lower-level evidence (category 2A) [4]. No randomized controlled trials (RCTs) have reported conclusive assessment results for RP or RT in men with PC, and the RCTs that have been conducted have had a small sample; randomization artifacts with imbalances in terms of health, age, and lymph or node assessment; and unclear or inconsistent risk classifications [9,10,11,12,13,14,15]. Clarity is lacking regarding whether RP or RT provides better biochemical failure–free survival (BFS), especially in healthy Relatively young men (life expectancy >10 years). Although a previous study demonstrated that RP is superior compared with RT for BFS in Relatively young men with PC [9], Relatively young men treated with high-dose conformal external-beam radiotherapy (EBRT), such as three-dimensional conformal radiotherapy (3D-CRT) or intensity-modulated radiotherapy (IMRT), appear to have excellent biochemical outcomes [16]. Therefore, we aimed to estimate the oncologic outcomes of RP or high-dose IMRT combined with short-term ADT for relatively young and relatively healthy men with NCCN-UIR-PC (life expectancy >10 years) by using head-to-head propensity score matching (PSM).

## 2. Patients and Methods

### 2.1. Database

We conducted a population-based cohort study using Taiwan National Health Insurance Research Data (NHIRD) linked to the TCRD. The TCRD was established in 1979 and contains almost 100% data of cancer cases in Taiwan [17]. The NHIRD includes all medical claims data on disease diagnoses, demographic characteristics, procedures, drug prescriptions, and enrollment profiles of all beneficiaries [18]. All patient identifiers were encrypted in the link of The NHIRD and TCRD. The Death Registry data are additionally linked to the NHIRD to verify the cause of death and vital status of each patient. The TCRD contains detailed information, such as AJCC stages, surgical procedures, techniques, RT, hormone treatments, and pathologic stages [19,20,21,22,23,24,25,26].

### 2.2. Study Cohort

The cohort comprised patients enrolled from the TCRD. We selected relatively young patients (≤65 years old) who had received a diagnosis of NCCN-UIR localized PC and received high-dose IMRT or RP between 1 January 2008, and 31 December 2016. We enrolled the relatively young men with UIR-PC with life expectancy >10 years in our cohort to receive standard RP or combination of IMRT and short-term HT based on NCCN guidelines. The index date was the date of PC diagnosis by pathologic conformed. Relatively young men with UIR-PC were followed up from the index date to 31 December 2018. Our protocols have been approved and reviewed by the Institutional Review Board of Tzu-Chi Medical Foundation (IRB109-015-B, date of approval 1 September 2020). Patient diagnoses were confirmed through a review of patients’ pathological data, magnetic resonance imaging of PC staging (cT1–T2c), pretreatment PSA levels (0–20 ng/mL), and grade group (1–3); steps were taken to ensure that patients who received a new diagnosis of localized PC and underwent RP or IMRT had no other cancer, clinical lymph node metastasis, or distant metastasis (DM). The RP type we examined was surgery to remove the entire prostate gland and the surrounding lymph nodes as treatment for men with localized PC [27]. Standard IMRT in our study involved prophylactic doses of 1.8 to 45 Gy per fraction to the pelvic lymph node and 54 Gy to the seminal vesicles as well as cone-down boosts of 72–81 Gy to cover the prostate (median dose: 75.6 Gy, median follow-up: 72.2 months). Insufficient doses of IMRT (<72 Gy) were excluded [12,13]. Relatively young patients with NCCN-UIR-PC who received IMRT without short-term (4–6 months) ADT were excluded. To prevent potential interference of all-cause death, we excluded relatively young patients with PC who did not receive standard RP or IMRT after PC diagnosis. For patients who received RP, we defined biochemical failure (BF) as a serum PSA level of ≥0.2 ng/mL according to the definition of BF by the AUA [28]. For patients who received IMRT, we defined BF as an increase in PSA of ≥2 ng/mL after PSA nadir in accordance with the Radiation Therapy Oncology Group-ASTRO Phoenix Consensus [29]. However, if BF was confirmed, then undergoing salvage radiation after RP; salvage prostatectomy, cryotherapy, or brachytherapy after IMRT; or systemic therapy such as ADT, advanced hormone therapy, target therapy, chemotherapy, and immune therapy did not disqualify patients from study inclusion. To compare their oncologic outcomes, such as all-cause death, BF, locoregional recurrence (LRR), and DM, patients who received RP and IMRT were assigned to groups 1 and 2, respectively. LRR or DM indicated clinically or radiologically overt local recurrence or distant failure. Prostate biopsy was performed for confirming local recurrence. A flow chart describing the cohort and the inclusion and exclusion criteria is drawn in Figure S2.

### 2.3. Study Covariates

Therapy type, age, diagnosis year, income, hospital area, hospital level (academic or nonacademic), clinical T-stage, grade group (max of Gleason grade), pretreatment PSA (ng/mL), and D’Amico classification, which might be associated with all-cause death, were used as our covariates. We scored comorbidities by applying the Charlson Comorbidity Index (CCI) [30,31], and well-known comorbidities associated with all-cause death. We only included comorbidities observed 6 months before and after the index date. Comorbid conditions in our study were included and verified according to the main International Classification of Diseases, Ninth Revision, Clinical Modification (ICD-9-CM) diagnostic codes for more than two repeated main diagnosis codes for visits to the outpatient department or the first admission. We also added the box plot to show comorbidities and CCI scores distribution for matched cohorts as Figure S1. Relatively young patients with few comorbidities who had CCI scores of >2 were excluded (relatively healthy, CCI ≤ 2) to enable comparisons of different curative-intent treatments for NCCN-UIR localized PC.

### 2.4. Endpoints

The primary endpoint was the risk of all-cause death between the patients underwent RP and high-dose IMRT combined with short-term ADT. Risk of BF, LRR, and DM between RP and high-dose IMRT combined with short-term ADT were secondary endpoints.

### 2.5. Propensity Score Matching

1:n matching can be used to increase precision in cohort studies [32]. The optimal matching in our study should be 1:4 ratio to reach the sufficient sample size in the analysis [32]. However, there were insufficient sample size to 1:4 ratio, we use a 1:2 ratio to increase sample size in our analysis. Of the 511 relatively young patients with NCCN-UIR-PC (Table 1), 318 and 193 belonged to the RP and high-dose IMRT groups, respectively. For the RP and IMRT groups, the mean follow-up durations after the index dates were 73.1 and 72.1 months (standard deviation = 17.5 and 18.3 months), respectively. No significant between-group differences after PSM were observed in any of the covariates, which included age, diagnosis year, CCI score, income, hospital area, hospital level, clinical T-stage, grade group, pretreatment PSA, and D’Amico classification (Table 1). The mean and median ages were balanced between the two groups (Table 1). As indicated in Table 1, no statistically significant differences (*p* > 0.05) in the variables were observed between the PSM cohorts. Most variables had a *p* value of >0.5, and even the *p* values of income, diagnosis year, CCI score, clinical T-stage, pretreatment PSA, and D’Amico risk classifications were close to 1, indicating that the distribution of matching variables was close. The outcomes of all-cause death, BF, LRR, DM, and follow-up duration were not matched because the survival time and oncologic outcomes were inconsistent between the treatment groups (Table 1).

### 2.6. Statistics

A Cox proportional hazards model was established to model the period from the index date to all-cause mortality with adjustment for confounders in relatively young men with NCCN-UIR localized PC. Head-to-head PSM was performed to decrease the effects of potential confounders during comparisons of treatment outcomes between the therapeutic groups. A width equal to 0.2 of the standard deviation of the logit of the propensity score have been matched the logit of the propensity score using calipers [33]. We selected controls with the same background covariates as the case participants to minimize any difference between the two groups [34]. All covariates in the high-dose IMRT group were matched at a 1:2 ratio with those in the RP group through PSM [35]. A robust sandwich estimator was used to account for clustering within matched sets and a Cox model was used to regress survival on treatment status [36]. We performed multivariable Cox regression analysis to calculate the hazard ratios (HRs) to determine whether factors such as therapy type, age, diagnosis year, CCI score, income, hospital area, hospital level, clinical T-stage, grade group, pretreatment PSA, and D’Amico classification might be necessary for double-adjustment to remove confounding if imbalance exists after PSM [37]. Potential prognostic factors were controlled in the analysis, and the endpoint was all-cause mortality in the treatment group.

In the multivariable Cox regression analysis, HRs were adjusted for treatments, diagnosis year, age, CCI score, income, hospital area, hospital level, clinical T-stage, grade group, pretreatment PSA, and D’Amico classification. Other secondary endpoints such as BF, LRR, and DM were estimated using a proportional subdistribution hazard regression model to overcome the competing risk of death in the analysis of time-to-event data [38,39]. We performed all analyses using SAS version 9.3 (SAS Institute, Cary, NC, USA). *p* < 0.05 was considered significant in a two-tailed Wald test. Risk of All-cause death was calculated for relatively young patients with NCCN-UIR-PC.

The risk of all-cause death was estimated using the Kaplan–Meier method, and differences among high-dose IMRT combined with short-term ADT or RP were determined using the stratified log-rank test to compare survival curves (stratified on matched sets) [40]. *p* < 0.05 was considered statistically significant.

## 3. Results

Therapeutic modality was a significant predictor of all-cause mortality after multivariable Cox regression analysis (Table 2). RP was associated with a higher OS than definitive high-dose IMRT combined with short-term ADT in Relatively young patients with NCCN-UIR-PC after multivariable Cox regression analysis. Because PSM was conducted well, there were no significant differences observed in the explanatory variables (Table 2). After multivariable Cox regression analysis, the adjusted HR (aHR) (95% confidence interval (CI)) of all-cause death for IMRT compared with RP was 2.76 (1.36–5.60, *p* = 0.0050). The aHR of BF for IMRT compared with RP was 2.74 (1.72–4.84, *p* < 0.0001) (Table 3). The aHR (95% CI) for significantly independent prognostic risk factors for LRR was 1.28 (1.09–1.90, *p* = 0.0324) for IMRT compared with that for RP (Table 4). The aHR for significantly independent prognostic risk factors for DM was 2.11 (1.40–4.88, *p* = 00052) for IMRT compared with that for RP (Table 5).

**Figure 1:**
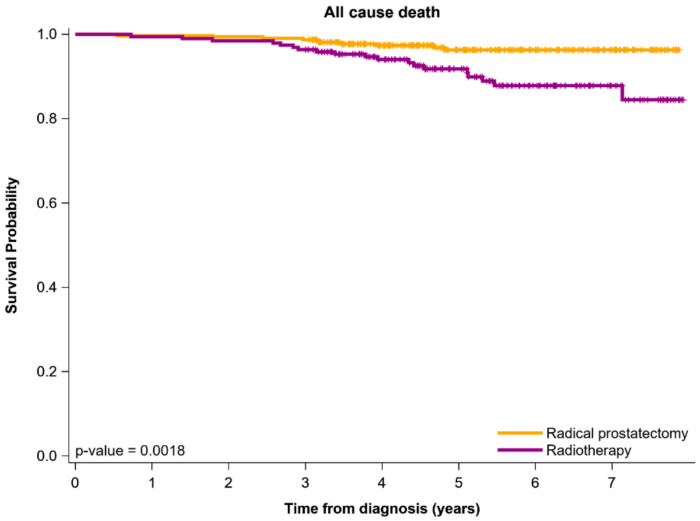
Overall survival curves obtained with the Kaplan–Meier method for propensity score–matched relatively young patients with unfavorable intermediate-risk prostate adenocarcinoma.

Figure 1 presents OS curves, which we obtained by using the Kaplan–Meier method for the PSM cohort of Relatively young men with NCCN-UIR-PC who received RP or high-dose IMRT combined with short-term ADT. The OS curve for RP was higher than that for definitive high-dose IMRT (at least 72 Gy, median dose of 75.6 Gy) in relatively young (age ≤ 65 years) patients with NCCN-UIR-PC. The 6-year OS rates for RP and IMRT were 96.9% and 90.2%, respectively. Moreover, we have showed the crude Kaplan–Meier survival curves of BFS, LRR-free survival, and DM-free survival as Figures S3–S5.

## 4. Discussion

No RCT has reported conclusive results from comparing the two primary treatments (high-dose IMRT and RP) for PC. Numerous failed trials have compared conventional RT and RP in the past [41,42], but only the ProtecT trial for patients with very low–risk or low-risk PC has completed accrual [43]. A phase III trial conducted by the National Cancer Institute of Canada Clinical Trials Group to compare active surveillance therapy with radical treatment rather than RT with RP in patients diagnosed as having favorable-risk PC trial is currently ongoing. Failed and completed accrual trials alike [14,15,41,42] have failed to present clear risk classifications or age effects for comparing oncological outcomes between RT and RP, especially through the use of contemporary RT techniques such as IMRT and RP in Relatively young patients with NCCN-UIR-PC.

Although oncologic outcomes following RP or RT have appeared to be independent of patient age [14,43,44], at least one study found a higher BF rate in relatively young patients treated with conventional doses of EBRT [9]. Although one study showed relatively young men with PC have poor outcomes in RT group compared with RP group [9], relatively young men treated with high-dose conformal EBRT such as IMRT appear to have excellent biochemical outcomes, overall survival [45], and fare as well as older patients [16].

The controversial oncologic outcomes of RP or RT in men with PC might be due to the relatively inhomogeneous risk groups of PC patients, broad distribution of patient age, and unclear multiple comorbidities strongly associated with patients’ life expectancy [9,14,16,43,44]. Clarity has been lacking regarding whether RT or RP is more suitable for relatively young patients with NCCN-UIR-PC, especially when contemporary RT techniques with a high irradiation dose combined with short-term ADT are used. For men with NCCN-UIR-PC, either RP or RT is the primary curative-intent treatment, and all recommendations in the NCCN guidelines are based on low-level evidence and consensus among urology experts (Category 2A). However, well-designed and reliable RCTs are lacking to support optimal treatments for patients with NCCN-UIR-PC based on age, health status, and comorbidities.

Some retrospective studies have shown that RT is an inferior treatment compared with RP for BFS in patients with PC, especially in relatively young men with intermediate- or high-risk PC [9,10]. Some retrospective studies have shown that neither RP nor RT is an independent predictor of BFS in patients with early T1–T2N0M0 PC [11,12,13], especially high-dose RT (≥72 Gy) [13]. However, a small RCT in Japan showed that in combination with endocrine therapy, either RP or EBRT with a low irradiation dose (<72 Gy) demonstrated favorable long-term outcomes for patients with locally advanced T2b–3N0M0 [14]. Another RCT conducted in 1982 demonstrated that RP was more effective than EBRT in establishing disease control [15], but it had a small sample, randomization artifacts with imbalances in health, age, and lymph node assessment, and outcomes worse than typical RT outcomes; hence, the results were never widely accepted [46]. In modern times, relatively young men with favorable intermediate-risk PC or UIR-PC treated with high-dose conformal EBRT (3D-CRT or IMRT) might have excellent biochemical outcomes; these biochemical results of high-dose RT were speculated to be compatible with the biochemical outcomes of RP, but no comparable data exists for RP and modern RT [16]. Therefore, determining whether RP or high-dose IMRT is more suitable for relatively young patients with NCCN-UIR-PC is crucial because of their relatively long life expectancy. Age effects and risk classifications for different treatments for these patients are unclear. Our study is the first to estimate the oncologic outcomes of RP or high-dose IMRT for patients with NCCN-UIR-PC who are relatively young and healthy.

All potential covariates related oncologic outcomes were matched well through PSM (Table 1). In addition, our study is the first to use NCCN risk classification and standard treatments from NCCN guidelines in relatively young patients with NCCN-UIR-PC. Nevertheless, the standard treatments followed by Taiwan physicians are based on low-level evidence and the consensus of urology experts (Category 2A) on the NCCN guidelines. Until now, no study with a sufficient sample size; satisfactory balance among patient health, age, lymph node assessment, and endocrine therapy variables; and sufficient irradiation dose administered using contemporary RT techniques has addressed the topic of which between RT and RP is optimal for treating men with NCCN-UIR-PC. Irrespective of the life expectancy estimated by the Social Security Administration tables, WHO’s Life Tables by country, or Memorial Sloan Kettering male life expectancy tool, the life expectancy of our enrolled patients was >10 years [4]. According to the NCCN guidelines, relatively young patients with NCCN-UIR-PC and fewer comorbidities (CCI = 0–2) could be treated with either RP or EBRT combined with short-term ADT (4–6 months) based on lower-level evidence and consensus from experts. However, as many studies have reported conflicting oncologic outcomes of using RP or RT [9,10,11,12,13,14,15], the optimal treatment for routine clinical practice remains unclear, especially for relatively young and healthy men.

Our study is the first study to report the different effects of RP and high-dose IMRT combined with short-term ADT on OS instead of focusing on BFS only, as previous studies have done [9,10,11,12,13,14,15]. After PSM, no significant difference in covariates was observed for all-cause death (Table 2), and no residual imbalance or residual confounding bias was observed, indicating that our PSM was satisfactory [37,47]. Therapeutic modality was an independent predictor factor of OS in relatively young and relatively healthy patients with NCCN-UIR-PC (Table 2). Our study is the first head-to-head PSM study to show that RP is superior to high-dose IMRT (≥72 Gy, median: 75.6 Gy) combined with short-term ADT in OS for relatively young and relatively healthy patients with NCCN-UIR-PC. In addition, we estimated the effects of the secondary endpoints of BF rate, LRR, and DM in these patients receiving RP or IMRT (Table 3, Table 4, Table 5). Furthermore, no significant differences in the covariates of BF, LRR, or DM were observed among our patients with NCCN-UIR-PC because no residual imbalance or residual confounding bias were present after PSM [37,47]. The only independent predictor for BF, LRR, and DM was the therapeutic modality (RP or IMRT) (Table 3, Table 4, Table 5). Our study is the first study to show the effect of RP or IMRT on OS for patients with PC; this is primarily because our study enrolled all NCCN-UIR-PC patients with a life expectancy of >10 years (relatively young and healthy) with a long follow-up time, which could overcome the competing risk of death [38]. In fact, for patients with a life expectancy of >10 years, considering differences in the therapeutic modalities used is crucial because the competing risk of death has a weak influence on relatively young and healthy patients, thus enabling determination of the true effect of different treatments on OS [38]. Conversely, for elderly men with multiple comorbidities, the competing risk of death is a highly influential factor that may obscure the evaluation of oncologic outcomes such as OS, LRR, DM, and all-cause death [38]. The assessment of OS, LRR, or DM requires a long follow-up time, during which time elderly patients with multiple comorbidities (life expectancy < 10 years) often die due to these comorbidities [38]. Estimation of the cumulative incidence of competing risks and competing risks regression would be done to estimate the accurate incidence and effects, because studies on elderly men in which a substantial number of participants are likely to die during long-term follow-up [38]. In old patients and guiding clinical decision-making between physicians and patients-self, a competing risk approach is crucial for accurately determining the disease risk for them [38]. Although men in our current study had a life expectancy of >10 years, the oncologic outcomes in our study were estimated using a proportional subdistribution hazard regression model to overcome the competing risk of death in the analysis of time-to-event data [38,39]. Taken together, our findings strongly suggest that RP, rather than high-dose IMRT combined with short-term ADT, should be the first choice of treatment for healthy relatively young men with NCCN-UIR-PC. This is the first study to evaluate the consensus (Category 2A) among experts on the NCCN guidelines and provide more conclusive guidance for deciding between RP and IMRT for treating men with NCCN-UIR-PC.

RP and RT each have their own advantages and disadvantages. The advantages of RT are effective long-term cancer control with high-dose treatments, less urinary incontinence compared with RP, and potential to cure patients of various ages and with significant comorbidities [16,43,44]. Therefore, RT could be reserved for elderly patients who have a serious comorbidity with NCCN-UIR-PC (life expectancy < 10 years), but it is unsuitable for relatively young and relatively healthy men (Table 2, Table 3, Table 4, Table 5 and Figure 1). RP may be a better treatment option for relatively young patients who want to avoid IMRT and who may be more comfortable monitoring their PSA level during the follow-up period rather than after IMRT. PSA naturally fluctuates at low but detectable levels during follow-up period [16]. Because there may be a small risk of radiation-induced malignancies in men undergoing radiotherapy for PC [48]. However, 15–20 more years of follow-up with modern approaches is necessary to quantify this risk further and determine whether this risk must be a clinical consideration for relatively young patients who select RT as their treatment for clinically localized PC [16]. Moreover, EBRT might increase the risk of late rectal symptoms compared with RP [49], and whether metastasis to the lymph nodes may occur cannot be determined with EBRT [43]. In addition, up to half of patients have some temporary bladder or bowel symptoms during RT [43,49,50]. Potential lymph node failure, late rectal toxicity, or small bowel toxicity might be troublesome for healthy relatively young men with NCCN-UIR-PC [43,50], thus, RP is more suitable for relatively young and relatively healthy men with NCCN-UIR-PC but is not suitable for all men with NCCN-UIR-PC. The disadvantages or contraindications of RP are high operative risks, neurogenic bladder, operative morbidity, and long-term incontinence [49]. However, for some patients, the lower risk of stress-induced urinary incontinence following high-dose RT compared with that following RP may be a crucial consideration for their quality of life [16]. Therefore, the choice between RT and RP for men with NCCN-UIR-PC depends on age and comorbidities.

We added the analysis table as Table S2 to show a table with significant predictors of all-cause death before PSM study. The significant poor predictors of all-cause death were IMRT, CCI ≥ 2, PSA ≥ 10, low incomes, non-medical centers, advanced T stages, and higher D’Amico classifications (Table S2). Table S1 lists the demographic and clinical characteristics of relatively young patients with unfavorable intermediate-risk prostate adenocarcinoma before PSM. The primary endpoint was the rate of all-cause death between the time points when the patients underwent RP and high-dose IMRT combined with short-term ADT. Therapy type, age, diagnosis year, income, hospital area, hospital level (academic or nonacademic), clinical T-stage, grade group (max of Gleason grade), pretreatment PSA, and advanced D’Amico classification, which might be associated with high risk of all-cause death. In addition, we have included the relatively young UIR-PC receiving ADT alone or IMRT alone in the Table S1. These is no PSM design in the Table S1, because the small sample size of UIR-PC receiving ADT alone or IMRT alone. Multivariate Cox proportional hazards regression model analysis of all-cause death was analyzed for these patients (RP, IMRT + short-term ADT, IMRT alone, and ADT alone) shown in Table S2.

Because our cohorts after PSM showed all covariates were very balance between RP and IMRT (Table 1). Thus, there was no residual imbalance in the multivariable Cox regression analysis after well-matched PSM [37,51]. Therefore, the multivariable Cox regression analysis did not identify any other covariates that were associated with death or PC progression after well-matched PSM and our study showed no residual imbalances after PSM in Table 2, Table 3, Table 4, Table 5. Although a table with significant predictors would be relevant, the well-matched PSM study without residual imbalances showing no significant predictors were correct [37,51]. PSM study would be mimic with the RCT and decrease the potential selection bias in the RP or IMRT [52,53]. If we did not choose PSM study, other selection bias related significant predictor would be produced. Our hypothesis is to estimate the oncologic outcomes of RP or high-dose IMRT combined with short-term ADT for relatively young and relatively healthy men with NCCN-UIR-PC (life expectancy > 10 years) instead of finding out the predictor factors. Therefore, PSM study design in the current study is feasible.

The strengths of this study are its sufficient sample size, long follow-up time, and the consistent covariates of relatively young and healthy men with NCCN-UIR-PC after PSM. In the current study, clinical characteristics were balanced between patients who underwent RP and those who underwent high-dose IMRT combined with short-term ADT. Most major covariates, such as age, diagnosis year, CCI score, income, hospital area, hospital level, clinical T-stage, grade group, pretreatment PSA, and D’Amico classification, were considered in our PSM analysis. This is the first head-to-head PSM study to estimate the effects of RP and high-dose IMRT with short-term ADT on OS, BF, LRR, and DM for relatively young and relatively healthy men with NCCN-UIR-PC. Our findings indicate that these patients with NCCN-UIR-PC obtain greater survival benefits from RP than from high-dose IMRT combined with short-term ADT. In the future clinical practice or prospective clinical trials, our findings would be a good reference for relatively young men with UIR-PC.

There were some limitations in our study. First, all relatively young men with UIR-PC enrolled in our study were Asian; therefore, there were unclear association of the corresponding ethnic susceptibility and our results should be cautiously extrapolated to Caucasian or Africa populations. However, until now, there has been no study of other races (African American, Caucasian, etc.) to estimate the oncologic outcomes of RP or high-dose IMRT combined with short-term ADT for relatively young and relatively healthy men with NCCN-UIR-PC (life expectancy > 10 years). There have been non-high-dose RT, non-IMRT, or not combination of IMRT with short-term ADT in the other studies. Thus, these studies were difficult to be compared with ours. Second, the diagnoses of all comorbid conditions in our study were dependent on ICD-9-CM codes. Nevertheless, the Taiwan Cancer Registry Administration often randomly reviews charts and interviews patients to verify diagnosis accuracy, and hospitals with outlier chargers or practices may be audited and heavily penalized if malpractice or discrepancies are identified. Third, RP techniques are improving with the development of open, laparoscopic, and robotic methods [21]. Modern surgical techniques were not analyzed in our study. However, in our study, regardless of whether conventional or modern techniques were employed, RP was superior to high-dose IMRT for treating relatively young and healthy men with NCCN-UIR-PC (Table 2, Table 3, Table 4 and Table 5). Thus, advancements in modern surgical techniques are unlikely to affect the conclusions of this research. Thus, to obtain real information on relatively young population specificity and disease occurrence, a large-scale randomized controlled trial comparing carefully selected relatively young population undergoing RP or high-dose IMRT is essential. Finally, the study does not contain information regarding dietary habits or body mass index, which may be risk factors for all-cause death. However, considering the statistical significance and the magnitude of the observed effects in our study, these limitations are unlikely to affect the study conclusions.

## 5. Conclusions

RP should be the first choice of treatment rather than high-dose IMRT combined with short-term ADT for relatively healthy relatively young men with NCCN-UIR-PC. After adjustment for confounders, RP was found to be superior to high-dose IMRT in terms of the patients’ overall survival, BF, LRR, and DM.

## Figures and Tables

**Table 1:** Propensity score-matched demographic and clinical characteristics of relatively young patients with unfavorable intermediate-risk prostate adenocarcinoma.

Variables		RP *n* = 318		IMRT *n* = 193		***p*-Value**
		*n*	(%)	*n*	(%)	
Age	Mean (SD)	61.8	(2.9)	61.8	(2.3)	0.6079
-	Median (IQR, Q1–Q3)	62	(58–64)	62	(59–64)	-
-	20–59	59	(18.6)	31	(16.1)	0.9641
-	60–65	259	(81.4)	162	(83.9)	-
Year of diagnosis	2011	55	(17.3)	37	(19.2)	0.9993
-	2012	59	(18.6)	34	(17.6)	-
-	2013	62	(19.5)	40	(20.7)	-
-	2014	73	(23.0)	42	(21.8)	-
-	2015	69	(21.7)	40	(20.7)	-
CCI scores	0	161	(50.6)	93	(48.2)	0.9623
-	1	85	(26.7)	53	(27.5)	-
-	2+	72	(22.6)	47	(24.4)	-
Income	<NTD$21,000	84	(26.4)	47	(24.4)	0.9093
-	NTD$21,000–NTD$30,000	104	(32.7)	67	(34.7)	-
-	NTD$30,000–NTD$45,000	73	(23.0)	51	(26.4)	-
-	NTD$45,000+	57	(17.9)	28	(14.5)	-
Hospital area	North	160	(50.3)	95	(49.2)	0.8864
-	Central	80	(25.2)	42	(21.8)	-
-	South	70	(22.0)	53	(27.5)	-
-	East	8	(2.5)	3	(1.6)	-
Hospital level	Medical center	201	(63.2)	108	(56.0)	0.5759
-	Other	117	(36.8)	85	(44.0)	-
Clinical T-stage	T1	17	(5.3)	12	(6.2)	0.9660
-	T2a	68	(21.4)	41	(21.2)	-
-	T2b	154	(48.4)	88	(45.6)	-
-	T2c	79	(24.8)	52	(26.9)	-
Grade group	1–2	5	(1.6)	10	(5.2)	0.4969
-	3	313	(98.4)	183	(94.8)	-
Pretreatment PSA (ng/mL)	Mean (SD)	9.2	(4.2)	9.5	(4.3)	0.5228
-	Median (Q1–Q3)	8.8	(6.0–11.7)	9.4	(6.2–12.0)	-
-	0–5	35	(11.0)	19	(9.8)	0.9904
-	5–10	149	(46.9)	87	(45.1)	-
-	10–20	134	(42.1)	87	(45.1)	-
D’Amico	Localized-low	117	(36.8)	67	(34.7)	0.9908
-	Localized-intermediate	100	(31.4)	60	(31.1)	-
-	Localized-high	77	(24.2)	51	(26.4)	-
-	Locally advanced	24	(7.5)	15	(7.7)	-
Follow-up time, months	Mean (SD)	73.1	(17.5)	72.2	(18.3)	-
All-cause death	-	10	(3.1)	19	(9.8)	0.0037
Biochemical recurrence	-	31	(14.7)	40	(20.7)	<0.0001
Locoregional recurrence	-	7	(2.2)	10	(5.2)	0.0061
Distant metastasis	-	9	(2.8)	18	(9.3)	0.0043

Abbreviations: CCI, Charlson Comorbidity Index; IMRT, intensity-modulated radiotherapy; IQR, interquartile range; PSA, prostate-specific antigen; Q: quartile; RP, radical prostatectomy; SD, standard deviation; T, tumor; NTD, New Taiwan dollar.

**Table 2:** Multivariable Cox proportional hazards regression model analysis of all-cause death in relatively young patients with unfavorable intermediate-risk prostate adenocarcinoma.

Covariates	Category	Adjusted HR *	(95% CI)	*p*-Value
Curative treatment	RP	Ref	-	0.0050
-	IMRT	2.76	(1.36–5.60)	-
Age	20–59	Ref	-	0.3907
-	60–65	1.59	(0.55–4.56)	-
Year of diagnosis	2011	Ref	-	0.3441
-	2012	1.07	(0.43–2.71)	-
-	2013	0.42	(0.14–1.23)	-
-	2014	0.56	(0.18–1.73)	-
-	2015	0.45	(0.11–1.85)	-
CCI scores	0	Ref	-	0.8774
-	1	0.79	(0.31–2.04)	-
-	2+	0.92	(0.28–2.99)	-
Clinical T-stage	T1	Ref	-	0.0915
-	T2a	1.07	(0.85–2.33)	-
-	T2b	1.76	(0.85–5.62)	-
-	T2c	1.14	(0.38–3.45)	-
Grade group (max Gleason grade)	1–2	Ref	-	0.7284
-	3	1.13	(0.56–2.28)	-
Pretreatment PSA (ng/mL)	0–5	Ref	-	0.4279
-	5–10	1.01	(0.52–1.22)	
-	10–20	1.09	(0.59–1.92)	
Income	<NTD$21,000	Ref		0.2333
-	NTD$21,000–NTD$30,000	0.87	(0.39–1.95)	-
-	NTD$30,000–NTD$45,000	0.82	(0.77–1.82)	-
-	NTD$45,000+	0.61	(0.33–1.76)	-
Hospital level	Medical center	Ref		0.5172
-	Others	1.26	(0.62–2.55)	-
Hospital area	North	Ref		0.9958
-	Central	1.03	(0.43–2.47)	-
-	South	1.07	(0.44–2.59)	-
-	East	1.24	(0.88–3.68)	-
Clinical T-stage	T1	Ref	-	0.2871
-	T2a	0.64	(0.24–1.68)	-
-	T2b	1.91	(0.69–12.36)	-
-	T2c	2.25	(0.41–3.78)	-
D’Amico	Localized-low	Ref	-	0.1190
-	Localized-intermediate/high	1.02	(0.42–1.12)	-
-	Locally advanced	1.12	(0.49–4.92)	-

Abbreviations: CCI, Charlson Comorbidity Index; CI, confidence interval; HR, hazard ratio; IMRT, intensity-modulated radiotherapy; PSA, prostate-specific antigen; Ref, reference group; RP, radical prostatectomy; T, tumor. * Covariates mentioned in Table 1 were adjusted for age, year of diagnosis, CCI score, income, hospital area, hospital level, clinical T-stage, grade group, pretreatment PSA, and D’Amico classification; NTD, New Taiwan dollar.

**Table 3:** Multivariable Cox proportional hazards regression model analysis of biochemical recurrence in relatively young patients with unfavorable intermediate-risk prostate adenocarcinoma.

Covariates	Category	Adjusted HR *	(95% CI)	*p*-Value
Curative treatment	RP	Ref	-	<0.0001
-	IMRT	2.74	(1.72–4.84)	-
Age	20–59	Ref	-	0.2723
-	60–65	1.01	(0.53–1.05)	-
Year of diagnosis	2011	Ref	-	0.7540
-	2012	1.41	(0.74–2.66)	-
-	2013	1.20	(0.63–2.29)	-
-	2014	1.03	(0.53–2.01)	-
-	2015	1.36	(0.73–2.52)	-
CCI scores	0	Ref	-	0.2526
-	1	1.04	(0.59–1.84)	-
-	2+	1.68	(0.86–3.28)	-
Clinical T-stage	T1	Ref	-	0.4324
-	T2a	1.05	(0.22–1.90)	-
-	T2b	1.09	(0.46–2.61)	-
-	T2c	1.53	(0.79–2.94)	-
Grade group (max Gleason grade)	1–2	Ref	-	0.1313
-	3	1.38	(0.91–2.11)	-
Pretreatment PSA (ng/mL)	0–5	Ref	-	0.5167
-	5–10	1.16	(0.51–2.26)	-
-	10–20	2.18	(0.63–3.92)	-
Income	<NTD$21,000	Ref	-	0.9229
-	NTD$21,000–NTD$30,000	1.02	(0.60–1.73)	-
-	NTD$30,000–NTD$45,000	1.09	(0.63–1.87)	-
-	NTD$45,000+	0.87	(0.44–1.72)	-
Hospital level	Medical center	Ref	-	0.2786
-	Others	1.27	(0.83–1.95)	-
Hospital area	North	Ref	-	0.2751
-	Central	1.16	(0.87–2.46)	-
-	South	1.25	(0.75–2.92)	-
-	East	1.30	(0.60–6.39)	-
Clinical T-stage	T1	Ref	-	0.5458
-	T2a	1.04	(0.49–1.44)	-
-	T2b	1.09	(0.41–1.98)	-
-	T2c	1.18	(0.40–2.16)	-
D’Amico	Localized-low	Ref	-	0.1444
-	Localized-intermediate/high	1.26	(0.41–2.26)	-
-	Locally advanced	2.08	(0.63–3.92)	-

Abbreviations: CCI, Charlson Comorbidity Index; CI, confidence interval; HR, hazard ratio; IMRT, intensity-modulated radiotherapy; PSA, prostate-specific antigen; Ref, reference group; RP, radical prostatectomy; T, tumor. * Covariates mentioned in Table 1 were adjusted for age, year of diagnosis, CCI score, income, hospital area, hospital level, clinical T-stage, grade group, pretreatment PSA, and D’Amico classification; NTD, New Taiwan dollar.

**Table 4:** Multivariable Cox proportional hazards regression model analysis of locoregional recurrence in relatively young patients with unfavorable intermediate-risk prostate adenocarcinoma.

Covariates	Category	Adjusted HR *	(95% CI)	*p*-Value
Curative treatment	RP	Ref	-	0.0324
-	IMRT	1.28	(1.09–1.90)	-
Age	20–59	Ref	-	0.2958
-	60–65	1.04	(0.27–1.49)	-
Year of diagnosis	2011	Ref	-	0.4456
-	2012	0.65	(0.22–1.92)	-
-	2013	0.66	(0.21–2.03)	-
-	2014	0.13	(0.02–1.11)	-
-	2015	0.78	(0.22–2.76)	-
CCI scores	0	Ref	-	0.6160
-	1	1.44	(0.60–3.46)	-
-	2+	1.08	(0.17–4.65)	-
Clinical T-stage	T1	Ref	-	0.4876
-	T2a	1.09	(0.31–1.67)	-
-	T2b	1.51	(0.70–2.47)	-
-	T2c	1.79	(0.37–3.31)	-
Grade group (max Gleason grade)	1–2	Ref	-	0.6298
-	3	1.02	(0.36–1.86)	-
Pretreatment PSA (ng/mL)	0–5	Ref	-	0.4772
-	5–10	1.14	(0.72–2.72)	-
-	10–20	2.07	(0.53–3.25)	-
Income	<NTD$21,000	Ref	-	0.4134
-	NTD$21,000–NTD$30,000	0.82	(0.70–1.81)	-
-	NTD$30,000–NTD$45,000	0.76	(0.52–1.75)	-
-	NTD$45,000+	0.43	(0.38–1.64)	-
Hospital level	Medical center	Ref	-	0.7466
-	Others	1.15	(0.49–2.69)	-
Hospital area	North	Ref	-	0.7801
-	Central	1.72	(0.82–2.00)	-
-	South	2.70	(0.91–3.04)	-
-	East	2.90	(0.89–4.10)	
Clinical T-stage	T1	Ref		0.5088
-	T2a	2.07	(0.79–5.45)	
-	T2b	1.30	(0.26–6.48)	
-	T2c	1.23	(0.47–3.25)	
D’Amico	Localized-low	Ref		0.4473
-	Localized-intermediate/high	1.95	(0.83–4.61)	
-	Locally advanced	2.59	(0.42–6.19)	

Abbreviations: CCI, Charlson Comorbidity Index; CI, confidence interval; HR, hazard ratio; IMRT, intensity-modulated radiotherapy; PSA, prostate-specific antigen; Ref, reference group; RP, radical prostatectomy; T, tumor. * Covariates mentioned in Table 1 were adjusted for age, year of diagnosis, CCI score, income, hospital area, hospital level, clinical T-stage grade group, pretreatment PSA, and D’Amico classification; NTD, New Taiwan dollar.

**Table 5:** Multivariable Cox proportional hazards regression model analysis of distant metastasis in relatively young patients with unfavorable intermediate-risk prostate adenocarcinoma.

Covariates	Category	Adjusted HR *	(95% CI)	*p*-Value
Curative treatment	RP	Ref	-	0.0052
-	IMRT	2.11	(1.40–4.88)	-
Age	20–59	Ref	-	0.7013
-	60–65	1.25	(0.40–3.87)	-
Year of diagnosis	2011	Ref	-	0.9648
-	2012	0.91	(0.31–2.64)	-
-	2013	0.89	(0.29–2.71)	-
-	2014	0.71	(0.22–2.29)	-
-	2015	0.68	(0.20–2.31)	-
CCI scores	0	Ref	-	0.4858
-	1	1.02	(0.89–1.90)	-
-	2+	1.05	(0.71–1.72)	-
Clinical T-stage	T1	Ref	-	0.7425
-	T2a	1.04	(0.64–4.27)	-
-	T2b	1.07	(0.73–7.57)	-
-	T2c	1.15	(0.37–3.58)	-
Grade group (max Gleason grade)	1–2	Ref	-	0.7957
-	3	1.11	(0.43–1.91)	-
Pretreatment PSA (ng/mL)	0–5	Ref		0.2498
-	5–10	1.05	(0.61–1.55)	-
-	10–20	1.27	(0.88–2.24)	-
Income	<NTD$21,000	Ref	-	0.1661
-	NTD$21,000–NTD$30,000	0.88	(0.52–2.14)	-
-	NTD$30,000–NTD$45,000	0.73	(0.25–2.13)	-
-	NTD$45,000+	0.52	(0.55–2.07)	-
Hospital level	Academic centers	Ref		0.1403
-	Nonacademic centers	0.80	(0.71–1.96)	-
Hospital area	North	Ref		0.1769
-	Central	1.38	(0.99–2.71)	-
-	South	1.60	(0.99–2.82)	-
-	East	2.03	(0.56–2.76)	-
Clinical T-stage	T1	Ref		0.3597
-	T2a	0.97	(0.38–1.44)	-
-	T2b	1.04	(0.37–2.83)	-
-	T2c	1.19	(0.42–2.25)	-
D’Amico	Localized-low	Ref		0.0981
-	Localized-intermediate/high	1.15	(0.46–1.86)	-
-	Locally advanced	1.72	(0.98–3.24)	-

Abbreviations: CCI, Charlson Comorbidity Index; CI, confidence interval; HR, hazard ratio; IMRT, intensity-modulated radiotherapy; PSA, prostate-specific antigen; Ref, reference group; RP, radical prostatectomy; T, tumor. * Covariates mentioned in Table 1 were adjusted for age, year of diagnosis, CCI score, income, hospital area, hospital level, clinical T-stage, grade group, pretreatment PSA, and D’Amico classification; NTD, New Taiwan dollar.

## Data Availability

Not applicable.

## Supplementary Materials

The following are available online at https://www.mdpi.com/2072-6694/13/7/1517/s1, Figure S1A. Box plot of propensity score distribution of radical prostatectomy and Intensity modulated radiotherapy before matching, Figure S1B. Box plot of propensity score distribution of radical prostatectomy and Intensity modulated radiotherapy after matching, Figure S2. Patient diagram for young patients (≤65 years old) with National Comprehensive Cancer Network unfavorable intermediate-risk localized prostate cancer, Figure S3. Biochemical recurrence-free survival curves obtained with the Kaplan–Meier method for propensity score–matched relatively young patients with unfavorable intermediate-risk prostate adenocarcinoma, Figure S4. Locoregional recurrence-free survival curves obtained with the Kaplan–Meier method for propensity score–matched relatively young patients with unfavorable intermediate-risk prostate adenocarcinoma, Figure S5. Distant metastasism-free survival curves obtained with the Kaplan–Meier method for propensity score–matched relatively young patients with unfavorable intermediate-risk prostate adenocarcinoma, Table S1. Before propensity score-matched Demographic and Clinical Characteristics of Young Patients with Unfavorable Intermediate-Risk Prostate Adenocarcinoma, Table S2. Multivariate cox proportional hazards regression model analysis of all-cause death in Young Patients with unfavorable intermediate-risk prostate adenocarcinoma before propensity scores matching.

## Abbreviations

PC	Prostate cancer
TCRD	Taiwan Cancer Registry database
NCCN	National Comprehensive Cancer Network
PSA	Prostate-specific antigen; AUA, American Urological Association
ASTRO	American Society for Radiation Oncology
UIR	Unfavorable intermediate risk
RP	Radical prostatectomy; RT, radiotherapy
ADT	Androgen-deprivation therapy; RCT, randomized controlled trial
3D-CRT	Three-dimensional conformal radiotherapy
IMRT	Intensity-modulated radiotherap
PSM	Propensity score matching
NHIRD	National health insurance research data
BF	Biochemical failure; LRR, locoregional recurrence
DM	Distant metastasis
ICD-9-CM	International Classification of Diseases, Ninth Revision, Clinical Modification
CCI	Charlson Comorbidity Index
OS	Overall survival
HR	Hazard ratio
EBRT	External-beam radiotherapy
CI	Confidence interval
BFS	Biochemical failure–free survival
aHR	Adjusted hazard ratio
