# Supplementary material for: Oncologic Outcomes of Radical Prostatectomy and High-Dose Intensity-Modulated Radiotherapy with Androgen-Deprivation Therapy for Relatively Young Patients with Unfavorable Intermediate-Risk Prostate Adenocarcinoma
Source: Cancers (Basel). 2021 Mar 25;13(7):1517. doi: 10.3390/cancers13071517 (PMC8036838; doi:10.3390/cancers13071517)
Supplement: Supplementary file 1 [file cancers-13-01517-s001.pdf]

cancers

Supplementary Materials: Oncologic Outcomes of Radical Prostatectomy and High-Dose Intensity-Modulated Radiotherapy with Androgen-Deprivation Therapy for Relatively Young Patients with Unfavorable Intermediate-Risk Prostate Adenocarcinoma

Szu-Yuan Wu, Shyh-Chyi Chang, Chang-I Chen and Chung-Chien Huang

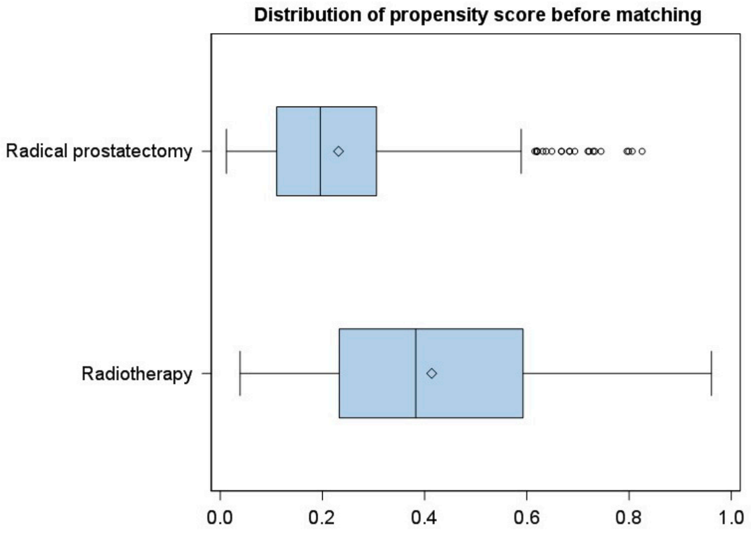

Figure S1A. Box plot of propensity score distribution of radical prostatectomy and Intensity modulated radiotherapy before matching.

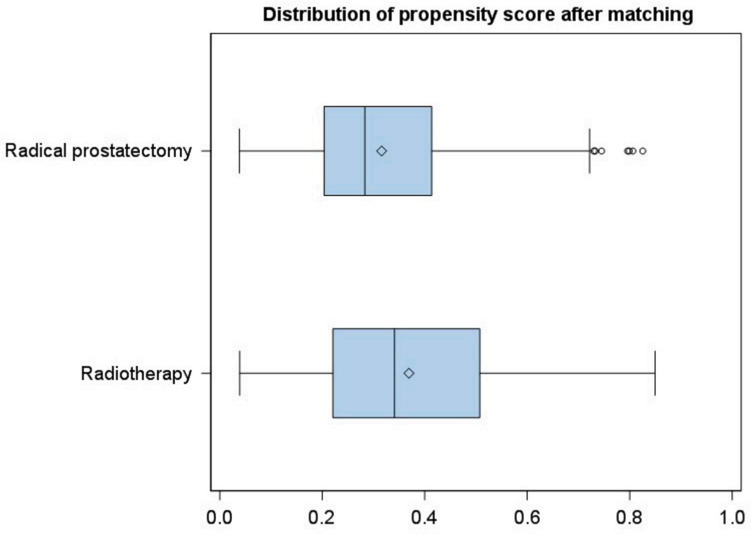

Figure S1B. Box plot of propensity score distribution of radical prostatectomy and Intensity modulated radiotherapy after matching.

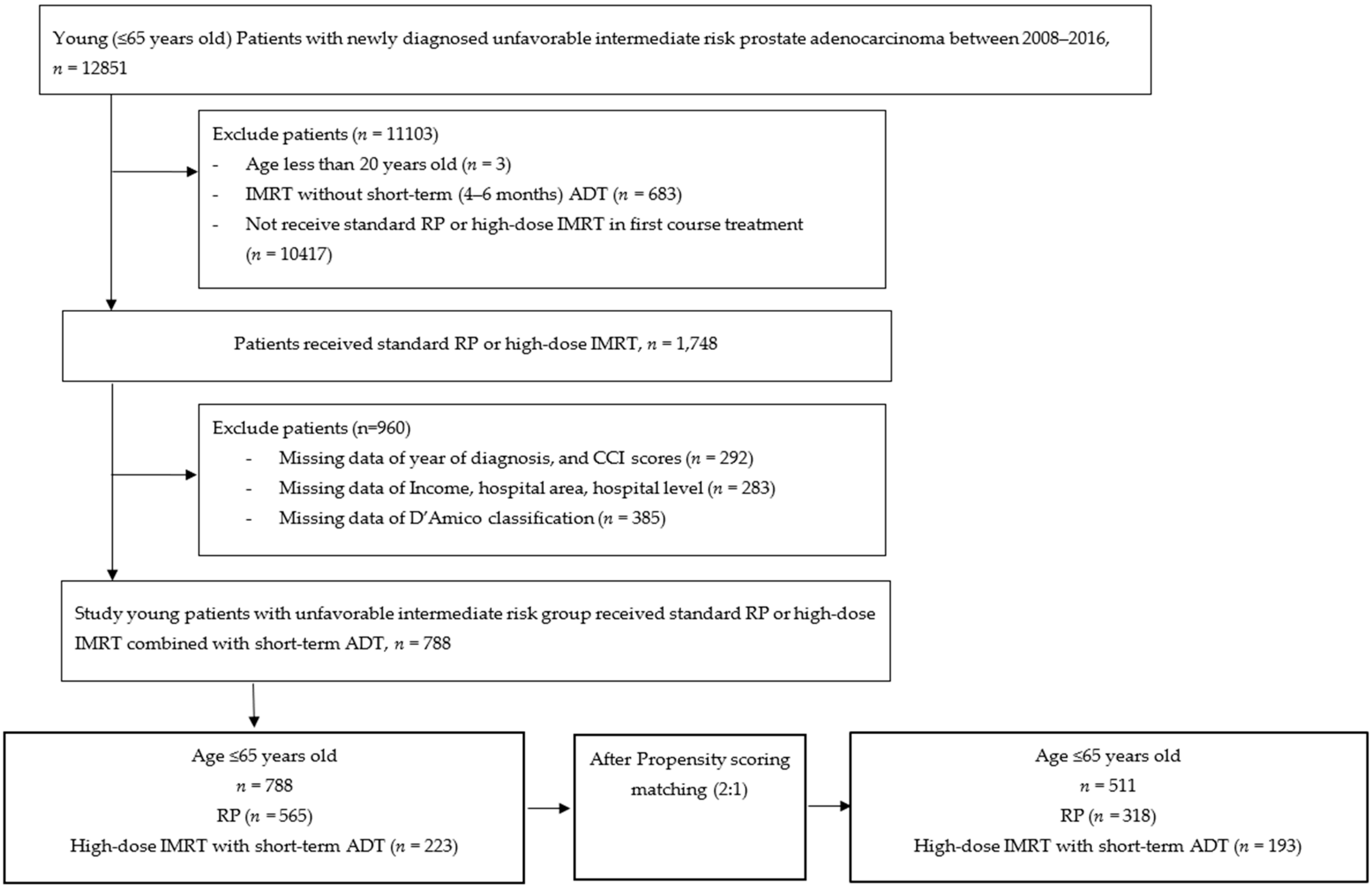

Figure S2. Patient diagram for young patients (≤65 years old) with National Comprehensive Cancer Network unfavorable intermediate-risk localized prostate cancer. CCI: Charlson Comorbidity Index; IMRT: intensity-modulated radiotherapy; RP: radical prostatectomy; ADT: androgen-deprivation therapy; *n*: number.

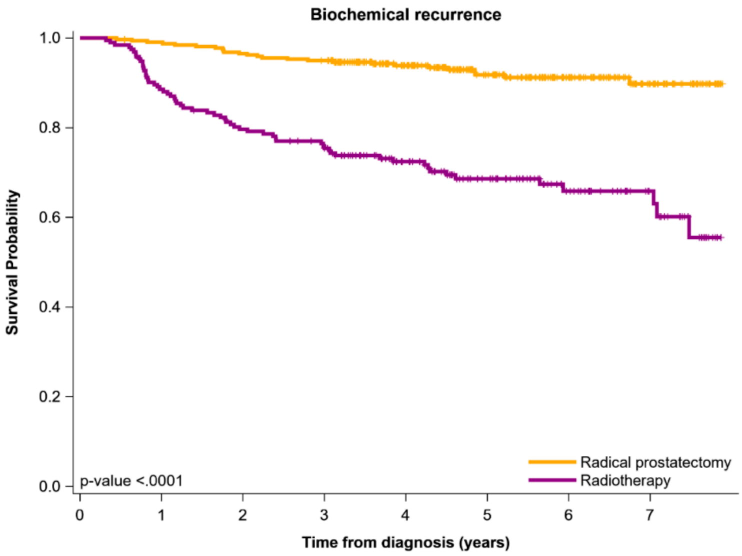

Figure S3. Biochemical recurrence-free survival curves obtained with the Kaplan–Meier method for propensity score–matched relatively young patients with unfavorable intermediate-risk prostate adenocarcinoma.

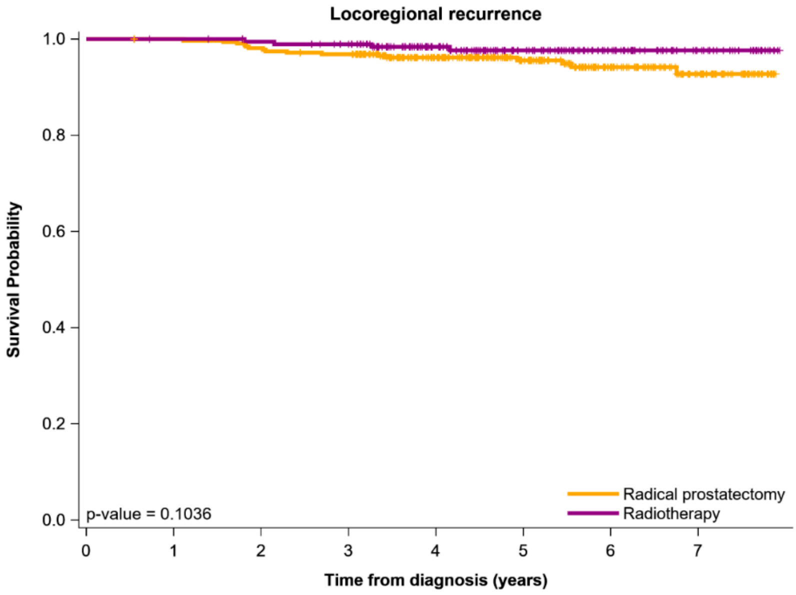

Figure S4. Locoregional recurrence-free survival curves obtained with the Kaplan–Meier method for propensity score–matched relatively young patients with unfavorable intermediate-risk prostate adenocarcinoma.

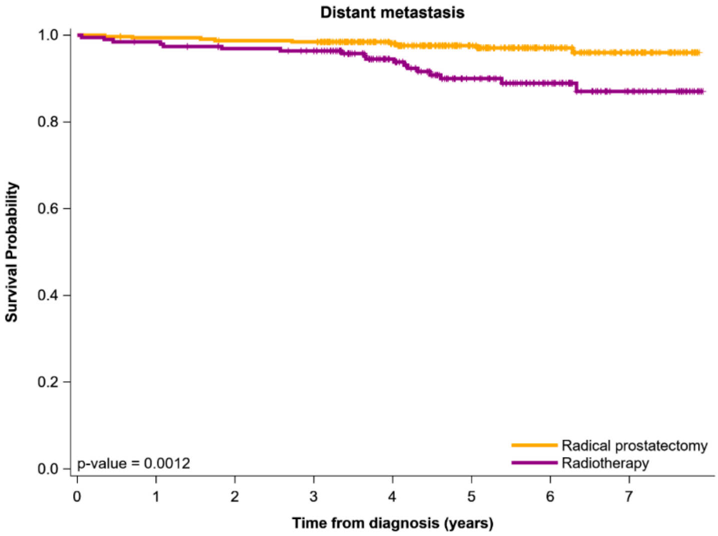

Figure S5. Distant metastasism-free survival curves obtained with the Kaplan–Meier method for propensity score–matched relatively young patients with unfavorable intermediate-risk prostate adenocarcinoma.

Table S1. Before propensity score-matched demographic and clinical characteristics of young patients with unfavorable intermediate-risk prostate adenocarcinoma.

Covariates		Before PS Matching						
		RP *n* = 565		IMRT+ADT *n* = 223		IMRT alone *n* = 97	ADT Alone *n* = 37	Type III Tests
		n	(%)	*n*	(%)	*n* (%)	*n* (%)	*p*-value
Age	Mean (SD)	61.6	(5.3)	64.1	(4.2)	64.2 (9.1)	64.4 (8.9)	<0.0001
-	Median (Q1–Q3)	63	(58–64)	64	(62-64)	64 (63–64)	64 (63–64)	
-	20–59	178	(31.5)	31	(13.9)	11 (11.3)	4 (10.8)	<0.0001
-	60–65	387	(68.5)	192	(86.1)	86 (88.7)	33 (89.2)	
Year of diagnosis	2011	84	(14.9)	44	(19.7)	22 (22.7)	9 (24.3)	<0.0001
-	2012	119	(21.1)	39	(17.5)	20 (20.6)	8 (21.6)	
-	2013	101	(17.9)	49	(22.0)	19 (19.6)	7 (18.9)	
-	2014	129	(22.8)	48	(21.5)	18 (18.6)	7 (18.9)	
-	2015	132	(23.4)	43	(19.3)	18 (18.6)	6 (16.2)	
CCI Scores	0	307	(54.3)	99	(44.4)	0 (0.0)	0 (0.0)	<0.0001
-	1	165	(29.2)	59	(26.5)	7 (7.2)	0 (0.0)	
-	2+	93	(16.5)	65	(29.1)	90 (92.8)	37 (100)	
Income	<NTD 21000	142	(25.1)	55	(24.7)	23 (23.7)	13 (35.1)	<0.0001
-	NTD 21000–30000	159	(28.1)	79	(35.4)	35 (36.1)	10 (27.0)	
-	NTD 30000–45000	106	(18.8)	60	(26.9)	26 (26.8)	9 (24.3)	
-	NTD 45000+	158	(28.0)	29	(13.0)	13 (13.4)	5 (13.5)	
Hospital area	North	300	(53.1)	104	(46.6)	34 (35.1)	8 (21.6)	<0.0001
-	Central	161	(28.5)	47	(21.1)	31 (32.0)	11 (29.7)	
-	South	91	(16.1)	69	(30.9)	30 (30.9)	15 (40.5)	
-	East	13	(2.3)	3	(1.3)	2 (2.0)	3 (8.1)	
Hospital level	Medical center	410	(72.6)	118	(52.9)	43 (44.3)	13 (35.1)	<0.0001
-	others	155	(27.4)	105	(47.1)	54 (55.7)	24 (64.9)	
Clinical T-stage	T1	257	(45.5)	99	(44.4)	43 (44.3)	12 (32.4)	<0.0001
-	T2a	128	(22.7)	49	(22.0)	26 (26.8)	13 (35.1)	
-	T2b	34	(6.0)	13	(5.8)	4 (4.1)	2 (5.4)	
-	T2c	146	(25.8)	62	(27.8)	24 (24.7)	10 (27.0)	
Grade group(max of Gleason grade)	1–2	5	(0.9)	12	(5.4)	8 (8.2)	1 (2.7)	<0.0001
-	3	560	(99.1)	211	(94.6)	89 (91.8)	36 (97.3)	
Preoperative PSA,(ng/mL)	0–5	123	(21.8)	25	(11.2)	19 (19.6)	7 (18.9)	<0.0001
-	5–10	292	(51.7)	90	(40.4)	29 (29.9)	9 (24.3)	
-	10–20	150	(26.5)	108	(48.4)	49 (50.5)	21 (56.8)	
D'Amico	Localized-Low	273	(48.3)	74	(33.2)	33 (34.0)	11 (29.7)	<0.0001
-	Localized-Intermediate/high	279	(49.4)	139	(62.3)	60 (61.9)	25 (67.6)	
-	-							
-	Locally advanced	13	(2.3)	10	(4.5)	4 (4.1)	1 (2.7)	
Follow-up time, months	Mean (SD)	74.5	(17.5)	73.7	(18.2)	70 (17.8)	64 (17.4)	
All-cause death	-	16	(2.8)	23	(10.3)	19 (19.6)	10 (27.0)	<0.0001
Biochemical recurrence	-	88	(15.6)	49	(22.0)	34 (35.1)	16 (43.2)	<0.0001
Locoregional recurrence	-	16	(2.8)	12	(5.4)	7 (7.2)	-	<0.0001
Distant metastasis	-	16	(2.8)	21	(9.4)	14 (14.4)	6 (16.2)	<0.0001

Abbreviations: CCI: Charlson Comorbidity Index; IMRT: intensity-modulated radiotherapy; IQR: interquartile range; PSA: prostate-specific antigen; Q: quartile; RP: radical prostatectomy; SD: standard deviation; T: tumor; ADT: Androgen deprivation therapy.

Table S2. Multivariate Cox proportional hazards regression model analysis of all-cause death in Young Patients with unfavorable intermediate-risk prostate adenocarcinoma before propensity scores matching.

Covariates	Category	Adjusted HR	(95% CI)	*p*-Value
Curative treatment	RP	ref	-	<0.0001
-	IMRT+ADT	1.78	(1.21–2.51)	-
-	IMRT alone	1.98	(1.42–3.87)	-
-	ADT alone	3.11	(2.01–4.42)	-
Age	20-59	ref	-	0.0363
-	60-65	1.83	(1.12–5.82)	-
Year of diagnosis	2011	ref	-	0.5941
-	2012	1.06	(0.71–2.90)	-
-	2013	0.98	(0.70–1.89)	-
-	2014	0.98	(0.72–1.92)	-
-	2015	0.77	(0.63–1.95)	-
CCI Scores	0	ref	-	0.0329
-	1	1.10	(0.83–1.41)	-
-	2+	1.45	(1.07–3.39)	-
Pretreatment PSA (ng/mL)	0–5	Ref	-	0.0108
-	5–10	1.19	(0.94–1.23)	-
-	10–20	1.97	(1.15–2.26)	-
Income	<NTD 21,000	ref	-	0.0115
-	NTD 21000-30000	0.869	(0.30–1.30)	-
-	NTD 30000-45000	0.49	(0.29–0.81)	-
-	NTD 45000+	0.43	(0.21–0.69)	-
Hospital level	Medical center	ref	-	0.0097
-	others	1.37	(1.14–1.99)	-
Hospital area	North	ref	-	0.7521
-	Central	1.23	(0.64–1.87)	-
-	South	1.27	(0.69–2.71)	-
-	East	1.71	(0.64–4.17)	-
Clinical T-stage	T1	ref	-	0.0047
-	T2a	1.16	(1.06–1.81)	-
-	T2b	1.96	(1.14–2.60)	-
-	T2c	2.28	(1.26–3.35)	-
D'Amico	Localized-Low	ref	-	0.0129
-	Localized-Inter/high	1.25	(1.18–1.89)	-
-	Locally advanced	2.72	(1.49–4.74)	-

Abbreviations: CCI:, Charlson Comorbidity Index; CI: confidence interval; HR: hazard ratio; IMRT: intensity-modulated radiotherapy; PSA: prostate-specific antigen; Ref: reference group; RP: radical prostatectomy; T: tumor.
